# Synonymous but not silent: functional codon bias reveals decoupled mitonuclear evolution in parasitic worms

**DOI:** 10.1093/bioadv/vbag105

**Published:** 2026-05-04

**Authors:** Kanhu Charan Das, Ruchishree Konhar, Devendra Kumar Biswal

**Affiliations:** Department of Zoology, North-Eastern Hill University, Shillong, Meghalaya, 793022, India; National Network Project @ Biodiversity Informatics Centre, North-Eastern Hill University, Shillong, Meghalaya, 793022, India; Department of Zoology, North-Eastern Hill University, Shillong, Meghalaya, 793022, India; National Network Project @ Biodiversity Informatics Centre, North-Eastern Hill University, Shillong, Meghalaya, 793022, India

## Abstract

**Motivation:**

The Codon usage bias, the preference for certain “silent” codons over others, is determined by a competition between two main forces: mutational pressure (random changes in DNA) and translational selection (the need for efficient protein production). However, we still lack a clear understanding of how this balance operates differently within the mitochondrial versus the nuclear genomes.

**Results:**

Here, we perform a comparative analysis of 120 parasitic helminths (Platyhelminthes and Nematoda), integrating mitochondrial genomes with matched nuclear transcriptomes to dissect the drivers of codon bias. Mitochondrial genes exhibit an AT-rich bias due to mutational factors, while nuclear codon preferences strongly correlate with gene expression, with highly-expressed genes utilizing optimal codons for efficiency. Across both compartments, patterns confirm pervasive selection acting on these seemingly “silent” sites, with codon optimization concentrated in the essential core oxidative phosphorylation (OXPHOS) subunits (like COX1, COX2) but relaxed in accessory genes. Crucially, comparative analyses revealed both mitonuclear coadaptation and, in some lineages, compartmental decoupling, demonstrating that parasites employ diverse evolutionary strategies to assemble their functional respiratory system, thus establishing that synonymous codons encode important functional adaptation relevant to parasite biology and control.

## 1 Introduction

Parasitic flatworms (Platyhelminthes) and roundworms (Nematoda) impose substantial burdens on human and animal health, agriculture, and wildlife. Schistosomes alone infect hundreds of millions globally, while soil-transmitted nematodes such as *Ascaris* and hookworms contribute to malnutrition and developmental impairment in endemic regions (Hotez *et al.* 2005, Caira and Littlewood 2013, Colley *et al.* 2014, Poinar 2015). Across these parasites, aerobic metabolism hinges on oxidative phosphorylation (OXPHOS) and therefore on efficient coordination between the mitochondrial and nuclear genomes that jointly encode the respiratory machinery (Lane and Martin 2010, Roger *et al.* 2017). Although mitonuclear co-dependence is a universal feature of eukaryotes, the translational-evolutionary strategies that underpin this coordination in helminths, particularly how synonymous sites are tuned within and between genomic compartments remain underexplored.

Synonymous codon-usage bias (CUB), the non-random use of synonymous codons arises from a balance between mutational processes and translational selection (Sharp and Li 1987, Bulmer 1991, Hershberg and Petrov 2008, Yang *et al.* 2021). Recent comparative work across animal models reinforces that codon usage covaries with expression, protein function, and effective population size, underscoring pervasive, lineage-specific selection on synonymous sites (Guschanski *et al.* 2021, Subramanian 2022). Animal mitochondrial genomes are compact, typically AT-rich, and shaped by strand-asymmetric replication and transcription that expose single strands to deamination, promoting A/T-ending codons independent of tRNA gene content (Perna and Kocher 1995, Saccone *et al.* 1999, DeSalle *et al.* 2017). By contrast, nuclear genes, especially highly expressed ones often track tRNA availability and ribosome dynamics, favouring codons that improve translational efficiency and accuracy (Akashi 1994, Duret and Mouchiroud 1999, Guschanski *et al.* 2021). Because OXPHOS complexes are chimeric, comprising subunits from both types of genomes, mitonuclear coevolution is expected to shape not only amino-acid replacements but also synonymous sites through effects on decoding speed, accuracy, and co-translational folding; disruption of this coupling can reduce respiratory performance and fitness (Rand *et al.* 2004, Ellison and Burton 2006, Burton and Barreto 2012, James *et al.* 2016).

Helminths provide a powerful comparative system to examine these dynamics. Molecular phylogenetics places Platyhelminthes within Spiralia and Nematoda within Ecdysozoa, implying deep divergence and potentially distinct translational constraints among orders (Blaxter *et al.* 1998, Dunn *et al.* 2008). Meanwhile, helminth mitogenomes have proliferated in public databases, and transcriptomic resources continue to rise in public repositories (Thornhill *et al.* 2023). Yet most prior work has treated mitochondrial and nuclear datasets in isolation, focused on few taxa, or relied on single metrics (e.g. RSCU or ENc) that cannot fully separate mutation from selection (Wright 1990, Peden 1999, Le *et al.* 2004, Chen *et al.* 2014). Consequently, we still lack an order-level, compartment-matched assessment of the mutation–selection balance at synonymous sites and of whether codon landscapes are coupled (co-adapted) or decoupled (compartment-specific) across parasitic lineages.

In this study, we assemble a mitonuclear dataset for 120 parasitic helminth species spanning Platyhelminthes and Nematoda and integrate complementary analyses to disentangle evolutionary forces shaping codon usage. We quantify codon preferences via Relative Synonymous Codon Usage (RSCU), assess departures from neutrality using Effective Number of Codons versus GC3s (ENc–GC3s) and neutrality plots of GC12 versus GC3, profile AT/GC nucleotide skews to capture strand-biased mutation, and evaluate gene-level Codon Adaptation Index (CAI) across the 13 canonical mitochondrial protein-coding genes. We further compare mitochondrial and matched nuclear codon landscapes using correlation-based approaches to test for mitonuclear concordance. The present study allows us to (i) resolve order-specific codon-usage signatures across compartments, (ii) quantify the extent to which selection, rather than mutation alone, shapes synonymous sites, and (iii) evaluate whether optimization concentrates in core OXPHOS subunits (e.g. COX1/COX2) relative to accessory genes (e.g. ATP8).

Our large-scale, compartment-matched analysis of 120 helminths establishes a general framework for how both intrinsic mutational pressures and extrinsic translational selection jointly shape these codon landscapes. We demonstrate that synonymous sites are neither silent nor random; their usage is functionally optimized, especially in core OXPHOS subunits, and is subject to either coordinated coadaptation or compartmental decoupling between the two genomes (Wolstenholme 1992, Lavrov and Pett 2016). This research clarifies helminth evolution, offers practical insights for codon optimization in functional assays, and outlines how mitonuclear constraints generate diverse evolutionary strategies across animal lineages, opening new perspectives for parasite control.

## 2 Methods

**Figure 1:**
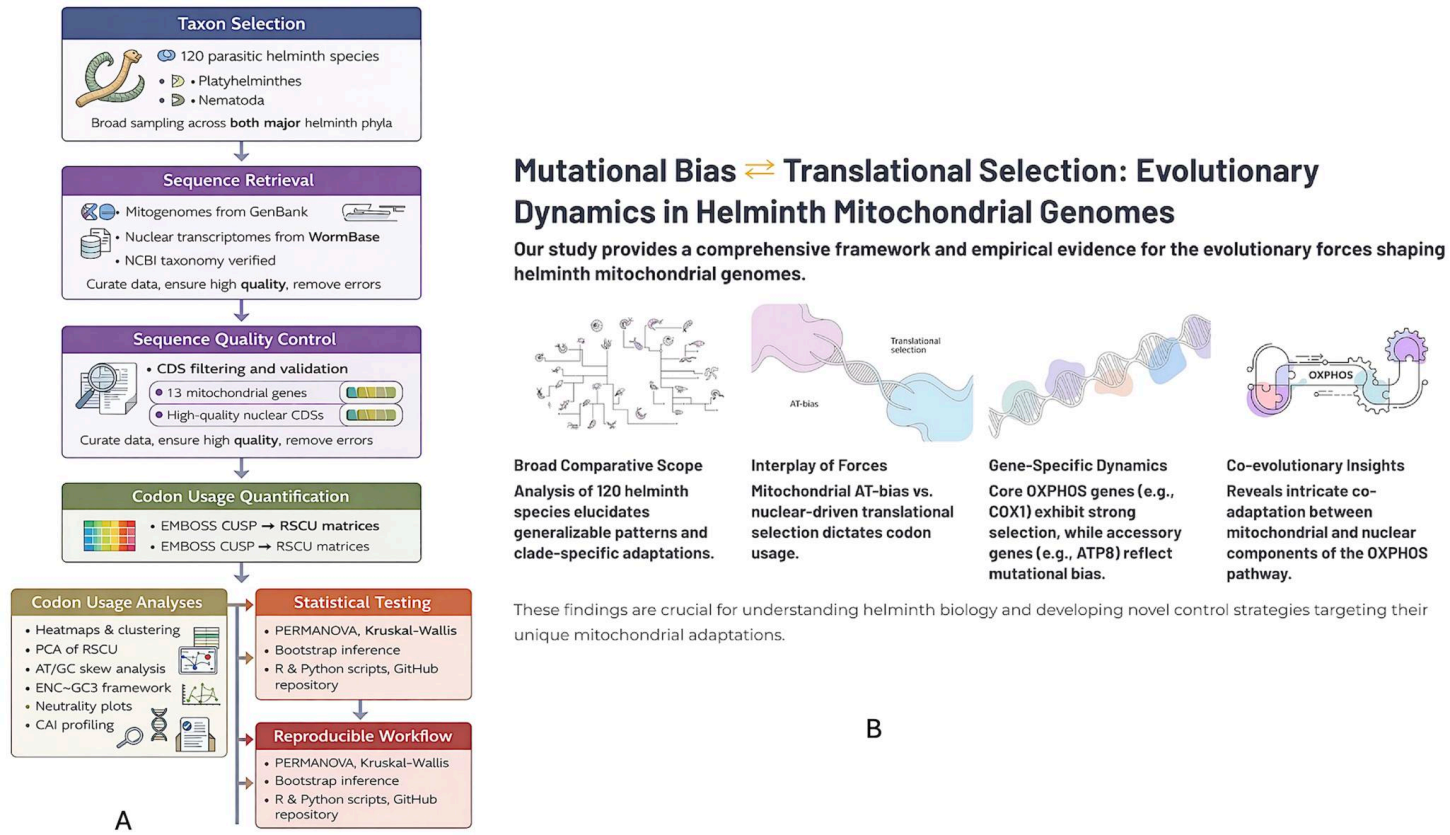
Analytical workflow and evolutionary framework of codon-usage analysis in parasitic helminths. (A) Computational pipeline used to analyse codon usage across 120 helminth species representing *Platyhelminthes* and *Nematoda*. Mitochondrial genomes and nuclear transcriptomes were retrieved from GenBank and WormBase, followed by CDS quality control and codon quantification using EMBOSS CUSP to generate RSCU matrices. Downstream analyses included heat maps and clustering, principal component analysis, nucleotide compositional bias (AT/GC skew), ENC–GC3 and neutrality plots, and Codon Adaptation Index profiling. Statistical testing and reproducible workflows were implemented in R and Python. (B) Conceptual framework illustrating how mutational bias and translational selection jointly shape codon-usage patterns in helminth mitochondrial genomes, revealing lineage-wide trends, gene-specific constraints in OXPHOS genes, and signatures of mitochondrial–nuclear co-evolution.

The overall analytical pipeline is illustrated in Fig. 1. Briefly, mitochondrial genomes and nuclear transcriptomes from 120 helminth species were retrieved, curated, and analysed using codon-usage metrics, compositional bias measures, and multivariate statistical approaches to investigate the evolutionary forces shaping codon usage.

### 2.1 Mitogenomes and transcriptome retrieval and annotation

We analysed 120 parasitic helminth species selected to maximize taxonomic breadth and ensure high data quality. The cohort intentionally includes representatives from both major helminth phyla: for Platyhelminthes, we sampled all three parasitic classes (Monogenea, Cestoda, and Trematoda) along with key free-living outgroups; for Nematoda, our sampling spanned all five major clades (I–V). Species were chosen based on the availability of high-quality, compartment-matched genomic resources specifically complete mitochondrial genomes and well-annotated nuclear transcriptomes, an approach analogous to established codon-usage studies utilizing RefSeq standards.

**Figure 2:**
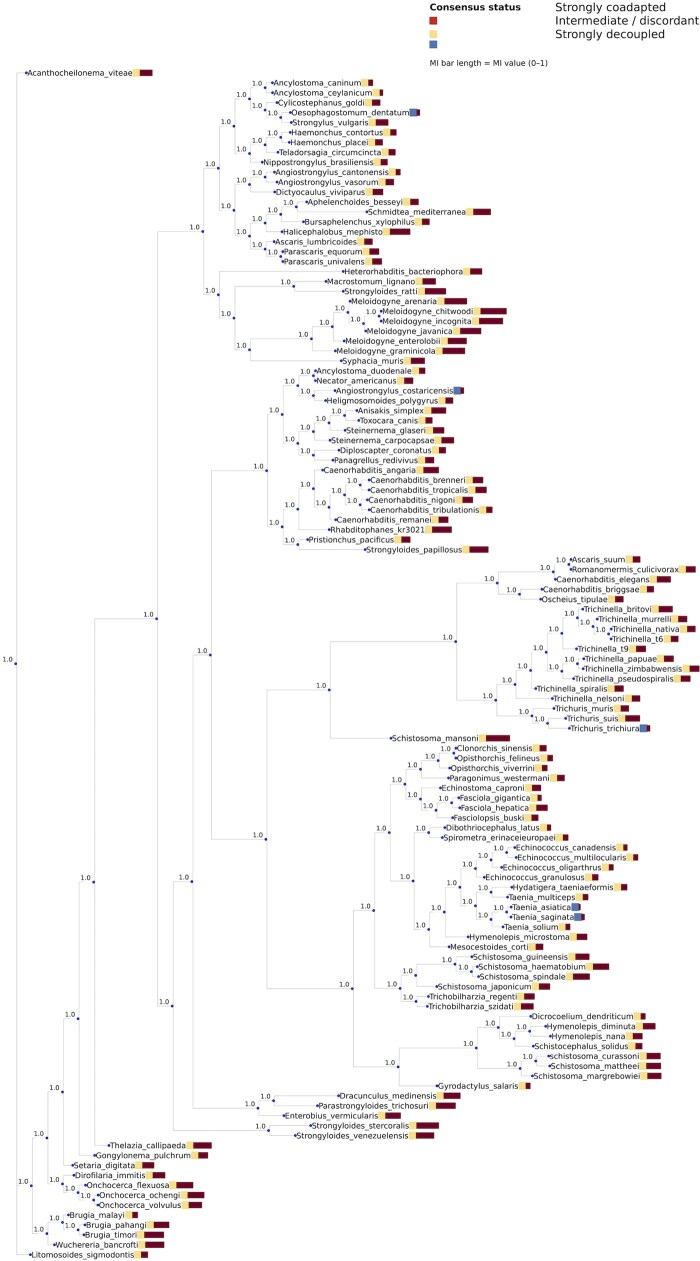
Phylogenetic diversity and mitonuclear coadaptation status across 120 helminth species. The consensus tree illustrates the evolutionary relationships among the sampled parasitic taxa, spanning major clades of Nematoda (I–V) and classes of Platyhelminthes (Monogenea, Cestoda, and Trematoda). This broad sampling strategy ensures that comparative analyses capture general codon-usage patterns across deep phylogenetic branches rather than lineage-specific idiosyncrasies. Coadaptation Status: Taxa are color-coded based on the statistical alignment of their mitochondrial and nuclear codon preferences: – Dark Red: Strongly coadapted clades where codon usage is highly coordinated between compartments. – Pink/Orange: Intermediate or discordant states. – Dark Blue: Strongly decoupled lineages exhibiting independent compartmental evolution. Mutual Information (Mi): The length of the horizontal bars represents the |M| value (0–1), quantifying the degree of dependence or information shared between mitochondrial and nuclear codon landscapes. Support Values: Node labels indicate consensus support (e.g. 1.0), providing a robust framework for interpreting subsequent principal component analysis (PCA) and clustering results.

To visually demonstrate this taxonomic coverage and provide a structural framework for interpreting our comparative results, we provide a phylogenetic tree of the 120 sampled taxa (Fig. 2), highlighting the deep evolutionary divergence and representation across both phyla. This broad sampling strategy ensures that our principal component analysis (PCA), clustering, and other comparative assessments capture general codon-usage patterns rather than lineage-specific idiosyncrasies, thereby enhancing the robustness and generality of our conclusions.

We assembled a comparative dataset of 120 parasitic helminths spanning Platyhelminthes and Nematoda. For each species we retrieved (i) the complete mitochondrial genome and corresponding coding sequences (CDSs) from NCBI GenBank (Sayers *et al.* 2025) and (ii) a matching nuclear transcriptome from WormBase (Sternberg *et al.* 2024) (http://wormbase.org/) (accession lists in Table S1). GenBank records were filtered to retain assemblies flagged as “complete mitochondrial genome.” Nuclear resources (genomes or transcriptomes) were taken from the most recent stable WormBase release available for each taxon. Taxonomic order assignments followed the NCBI Taxonomy backbone (Federhen 2012).

### 2.2 Sequence preparation and quality control

For each mitogenome, we extracted the 13 canonical protein-coding genes (PCGs: ATP6, ATP8, COX1–3, COB, NAD1–6, NAD4L) using the GenBank feature table and verified open reading frames under the appropriate mitochondrial genetic code reported in the record (typically NCBI translation table 5, “invertebrate mitochondrial”). CDSs containing internal stop codons after translation, frameshifts, or >1% ambiguous nucleotides (N) were excluded. Nuclear datasets were processed by extracting all CDSs from the reference GFF/GTF or WormBase CDS FASTA; sequences <150 bp or with >1% ambiguous sites were removed. For downstream analyses requiring species-level summaries (e.g. skews, ENc–GC3s, neutrality), CDSs were concatenated within species by compartment.

### 2.3 Codon usage profiling and heat map visualization

For each species and compartment (mitochondrial, nuclear), raw codon counts were obtained with EMBOSS CUSP (v6.6.0) from the curated CDS sets (Rice *et al.* 2000). Stop codons were excluded, and sequences with internal stops or >1% ambiguous bases were removed during QC (see Sequence preparation). Counts were converted to Relative Synonymous Codon Usage (RSCU) within species,


$$\text{RSC}U_{\text{ij}}=\frac{X_{ij}}{\frac{1}{n_{i}}\sum_{j=1}^{n_{i}}X_{ij}}$$

where *X_ij_* is the count of codon *j* for amino acid *i* with n_i synonymous codons (Sharp and Li 1987). We assembled a species-by-codon matrix (120 × 60 RSCU values; ATG and all stop codons removed) for each compartment. For visualisation, RSCU values were centered and scaled by codon (z-scores) to emphasise relative preferences across taxa. Heat maps were generated in Python (pandas/numpy/matplotlib) with codons arranged by amino acid and species ordered by taxonomic order; where indicated, rows were hierarchically clustered (Euclidean distance, complete linkage) to highlight lineage-specific structure. In the heat map, columns represent sense codons and rows represent species; cell intensity encodes the scaled RSCU, with lower-than-expected usage shown as cool tones and higher-than-expected usage as warm tones. This representation provides a compact overview of conserved versus order-specific codon preferences across mitogenomes and matched nuclear transcriptomes. All intermediate CSVs (codon counts and RSCU matrices) and plotting scripts are available in the project repository for full reproducibility.

### 2.4 Principal component analysis of codon usage patterns

For each species and compartment (mitochondrial, nuclear), we converted codon counts (EMBOSS CUSP **o**utput) to relative synonymous codon usage **(**RSCU**)** following Sharp and Li (1987):


$$\text{RSC}U_{\text{ij}}=\frac{X_{ij}}{\frac{1}{n_{i}}\sum_{j=1}^{n_{i}}X_{ij}}$$

where *X_ij_* is the count of codon *j* for amino acid *i* and n_i is the number of synonymous codons for i. By definition, RSCU = 1 indicates equal use among synonyms; values >1 reflect preferential use (strong preference often taken as RSCU ≥1.5) (Sharp and Li 1987, Huo *et al.* 2021). We assembled 120 × 60 RSCU matrices per compartment (stop codons TAG/TAA/TGA and the start codon ATG excluded). To emphasise among-species differences, RSCU values were centred and scaled by codon (z-scores). Principal component analysis (PCA) was performed in R (v4.3) using FactoMineR (v2.8) and visualised with factoextra (v1.0.7) (Lê *et al.* 2008, Kassambara and Mundt 2020). We inspected codon loadings (contrib, cos^2^) to identify synonymous triplets driving separation along the first two components and produced biplots with 95% confidence ellipses for taxonomic orders. To evaluate structure statistically, we tested order-level clustering on scaled RSCU using PERMANOVA (10 000 permutations; Euclidean distance; vegan v2.6–4) with Benjamini–Hochberg correction for multiple comparisons (Benjamini and Hochberg 1995, Anderson 2001). All matrices, scripts, and figure code are provided in the project repository for full reproducibility.

### 2.5 Nucleotide compositional bias: AT skew and GC skew analysis

Strand-asymmetric base composition was quantified on concatenated CDS sets from the mitochondrial and nuclear compartments. CDSs were analysed in the coding orientation (minus-strand genes reverse-complemented), and sequences containing internal stops or >1% ambiguous bases (N) were removed (see Sequence preparation). Counts of A, T, G and C were used to compute AT-skew and GC-skew (Perna and Kocher 1995):


$$\text{AT-skew}=\frac{A-T}{A+T},\;\text{GC}-\text{skew}=\frac{G-C}{G+C}$$

Positive values indicate enrichment of the first base in each pair on the coding strand; negative values indicate enrichment of the second. Skews were calculated at the species level and then summarised by taxonomic order. All calculations were implemented in Python 3.10 (pandas v2.1, NumPy v1.26). To visualise lineage differences, AT-skew (y-axis) was plotted against GC-skew (x-axis) for every species, with order centroids and 95% bootstrap ellipses overlaid (10 000 resamples of genes within species). Because strand asymmetries often reflect replication-linked deamination and transcriptional exposure (Perna and Kocher 1995, Lobry 1996, Xia 2012), dispersion among orders in this plane is interpreted as variation in underlying mutational regimes and genome architecture rather than sampling noise. Scripts and per-species skew tables are available in the project repository.

### 2.6 Codon-usage analysis: ENC, GC3 and neutrality plots

Analyses were performed on mitochondrial and nuclear CDSs from 120 helminth species, with results summarised by taxonomic order. CodonW v1.4.4 was used to compute the effective number of codons (ENC) and GC content at third positions (GC3) for each gene (Wright 1990, Peden 1999). ENC quantifies overall codon bias (20 = maximal bias; 61 = none) and, when examined against GC3, helps distinguish mutational from selective components of bias (Wright 1990, Hershberg and Petrov 2008).

#### 2.6.1 ENC–GC3 framework

For each gene, ENC was plotted against GC3 and compared to Wright’s neutral expectation,


$$\text{EN}C_{\;\text{exp}\;}=2+{\text{GC}}_{3}+\frac{29}{{\text{GC}}_{3}^{2}+{\left(1-{\text{GC}}_{3}\right)}^{2}}$$

where departures below the curve indicate codon bias stronger than predicted by nucleotide composition alone (Wright 1990). Genes shorter than 100 codons were excluded.

#### 2.6.2 Neutrality (GC12 vs GC3)

To assess the mutation-selection balance directly, we computed GC at each codon position using Python 3.10 (pandas/numpy). Mean GC at the first two positions was defined as


$$\text{GC}12=\frac{\text{GC}1+\text{GC}2}{2}$$

We regressed GC12 on GC3 for each compartment; a slope ≈1 implies mutation-driven composition, whereas a slope ≪1 suggests selective constraints at the first/second positions uncoupling them from third-position variation (Sueoka 1995).

#### 2.6.3 Comparative summaries and plotting

Order-level differences were evaluated on gene-wise metrics using Kruskal–Wallis tests with Benjamini-Hochberg correction (Benjamini and Hochberg 1995). Figures were generated in matplotlib v3.8. Together, ENC-GC3 and neutrality analyses provide complementary evidence on whether codon usage reflects primarily background mutational pressure or selection on translation (Wright 1990, Hershberg and Petrov 2008).

### 2.7 Codon Adaptation Index (CAI) profiling

The Codon Adaptation Index (CAI) is a widely used measure of synonymous codon usage bias, defined as the geometric mean of the relative adaptiveness values of the codons in a gene (Sharp and Li 1987). In practice, CAI quantifies how closely a gene’s codon usage pattern matches a reference set of highly expressed genes. CAI values range from 0 to 1, where 1 indicates that a gene exclusively uses the optimal (most frequent) codons for each amino acid (as found in the reference set), and lower values indicate a lesser degree of adaptation to the reference codon usage pattern (Sharp and Li 1987). This index is biologically relevant as it reflects the extent to which natural selection may have shaped codon usage to enhance translational efficiency or accuracy in protein-coding genes.

For each of the 120 species in our dataset, we analyzed the 13 canonical mitochondrial protein-coding genes—ATP6, ATP8, COX1, COX2, COX3, COB, NAD1, NAD2, NAD3, NAD4, NAD4L, NAD5, and NAD6 to profile codon usage bias. We calculated the CAI for each gene using the program CodonW (version 1.4.x; Peden 1999), which was executed through a Python script to automate batch processing. CodonW computes CAI by first determining the relative adaptiveness (w_i_) of each codon (the ratio of that codon’s usage to the usage of the most frequent synonymous codon for the same amino acid in the reference set) and then taking the geometric mean of these w values across all codons in the gene (Sharp and Li 1987). In the absence of an external reference genome, we used each species’ own set of mitochondrial protein-coding genes as the reference set for that species’ CAI calculation (i.e. optimal codons were inferred from the pooled codon usage of the 13 genes in the mitochondrial genome). All CAI results were compiled into a matrix of size 120 (species) × 13 (genes), and this matrix was saved as a CSV file for downstream analysis.

It should be noted that accurate estimation of CAI ideally requires a well-defined set of highly expressed genes to serve as the reference standard for codon preference (Sharp and Li 1987). In our case, a reliable expression-based reference could not be obtained from the available transcriptomic data. As a consequence, the CAI values reported here reflect relative codon adaptation within each mitochondrial genome rather than an absolute measure of expression optimization. This approach is appropriate for comparing codon usage bias among the mitogenomic genes of these species, but the absence of a true expression-calibrated reference set is a limitation to bear in mind when interpreting the CAI results.

### 2.8 Statistical testing and reproducibility

All statistical analyses were performed in R (R Core Team 2023) and Python. Analyses were performed in R (v4.3.x) and Python (v3.10). Exact scripts, parameters, and package versions (including vegan v2.6–4, FactoMineR v2.8, factoextra v1.0.7, pandas v2.1, numpy v1.26, matplotlib v3.8) are archived in our GitHub repository (https://github.com/bicnehu/mito-transcriptome-codon-analysis) (06 March 2026, date last accessed). Random seeds were fixed [set.seed(12345) in R; PYTHONHASHSEED = 0] and environment files (renv.lock, requirements.txt) are provided to ensure full reproducibility.

**Order-level clustering:** We tested whether codon-usage profiles cluster by taxonomic order using PERMANOVA on Euclidean distances of z-scored RSCU matrices (adonis2, 10 000 permutations). We report pseudo-F, R^2^, and permutation *P*-values, and verified homogeneity of dispersion with betadisper/permutest (Anderson 2001, Oksanen *et al.* 2022).

**Neutrality and ENC-GC3 analyses:** Slopes of GC12 ∼ GC3 were estimated by ordinary least squares with 10 000 nonparametric bootstrap resamples of genes within species to obtain 95% CIs. ENC–GC3 deviations from Wright’s neutral expectation were summarized per species and compared among orders with Kruskal-Wallis tests. **Multiple testing:** *P*-values from omnibus and pairwise contrasts were adjusted using the Benjamini-Hochberg false-discovery rate procedure (Benjamini and Hochberg 1995). This pipeline provides end-to-end reproducibility for sequence retrieval, codon-usage quantification, and all statistical inferences.

## 3 Results

### 3.1 Genome retrieval and composition

Complete mitochondrial genomes (*n* = 120) and their corresponding coding‐sequence (CDS) files were downloaded from the NCBI repository. In parallel, transcriptome assemblies for these same 120 parasitic taxa were obtained from WormBase (http://wormbase.org/) (06 March 2026, date last accessed). Each mitogenome and transcriptome dataset comprised both coding and non-coding regions. The coding regions included 13 canonical mitochondrial protein-coding genes (PCGs): ATP6, ATP8, COX1, COX2, COX3, COB, NAD1, NAD2, NAD3, NAD4, NAD4L, NAD5, NAD6 as well as the full complement of tRNA and rRNA genes.

### 3.2 Codon usage and translational efficiency in parasitic species

#### 3.2.1 Heat map of codon usage bias in genomes

**Figure 3:**
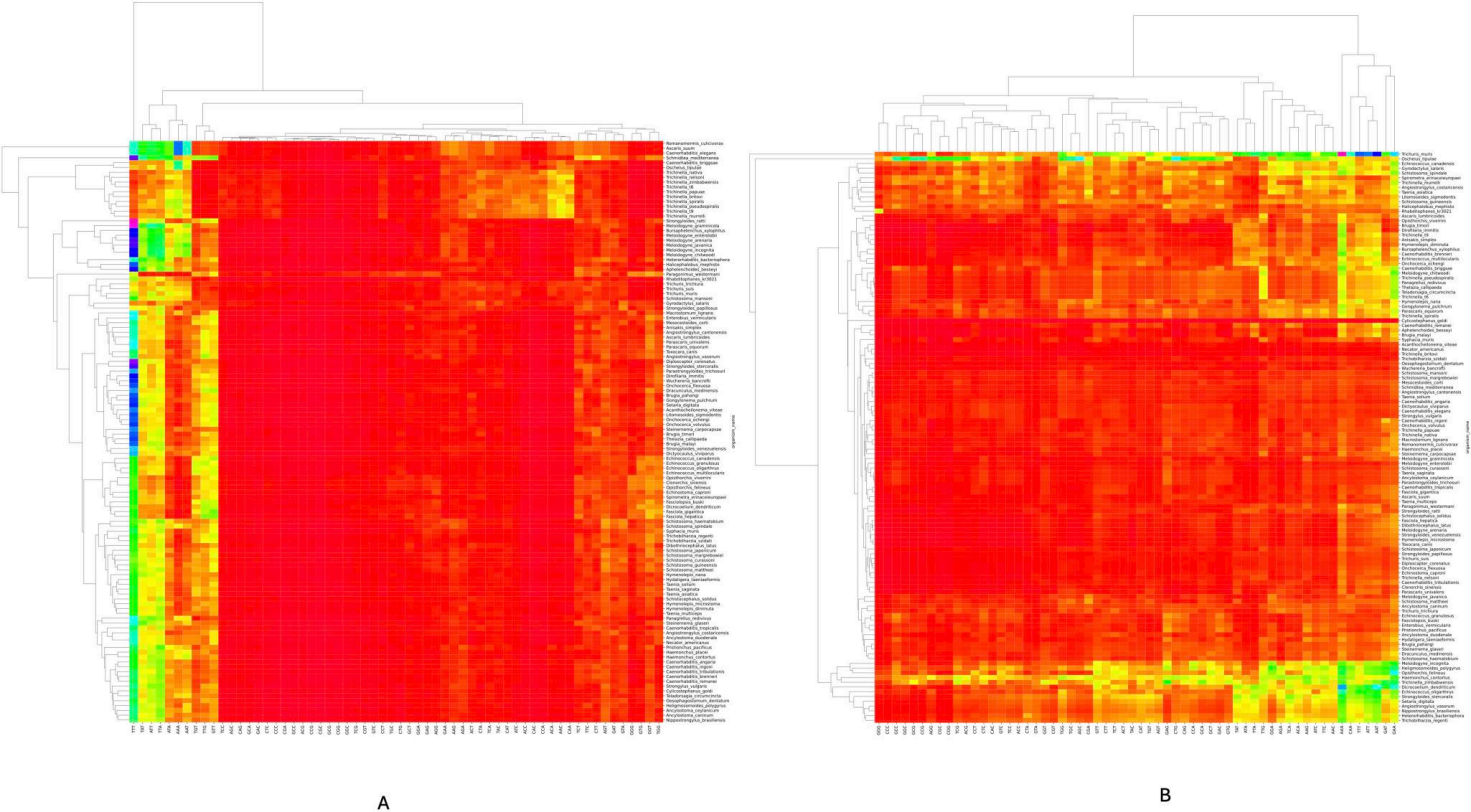
Heat maps of relative synonymous codon usage (RSCU) across 120 parasitic helminths. (A) Mitochondrial genomes and (B) nuclear transcriptomes. Rows represent species and columns represent the 61 sense codons. Cell colours correspond to RSCU values, with red indicating under-represented codons (RSCU < 1), yellow indicating approximately neutral usage (RSCU ≈ 1), and magenta indicating over-represented codons (RSCU > 1). The colour gradient spans the observed quantitative range of RSCU values across datasets, enabling direct comparison between mitochondrial and nuclear codon usage patterns. Hierarchical clustering of both taxa and codons highlights lineage-specific codon usage signatures across parasitic helminths. Enrichment of AT-rich codons (e.g. TTT, AAA, ATT) illustrates the pervasive AT compositional bias and its contribution to lineage-specific codon preferences.

A codon-usage bias (CUB) heat map was generated for all 120 mitogenomes and associated transcriptomes (Fig. 3A and B). The analysis reveals a highly conserved codon-usage profile across taxa, indicating that CUB does not drive major taxonomic differentiation. Both datasets exhibit a pronounced A/T–rich codon preference over G/C–rich codons. The most frequently employed codons in both mitochondrial and transcriptomic sequences are TTT (Phe), AAA (Lys), ATT (Ile), AAT (Asn), and TTG (Leu). Mitochondrial genomes further show elevated usage of TTA (Leu), TAT (Tyr), GTT (Val), and ATA (Ile), whereas transcriptomes preferentially utilize GAA (Glu), GAT (Asp), CAA (Gln), ATG (Met), and AAG (Lys). In the heat map, codons are arrayed along the X-axis and species along the Y-axis; cell coloration transitions from red (low relative usage) to deep magenta (high relative usage), quantifying the degree of bias.

#### 3.2.2 RSCU analysis and PCA of codon usage patterns across parasitic orders

Relative synonymous codon usage (RSCU) values were calculated for 120 parasitic mitochondrial genomes and their corresponding transcriptomes. These RSCU matrices were then subjected to principal component analysis (PCA) to elucidate underlying patterns in codon usage.

In the mitochondrial dataset, the first two principal components accounted for 49.6% (PC1) and 17.7% (PC2) of the total variance, yielding a cumulative explanation of 67.3% (Le *et al.* 2004). PCA revealed a pronounced clustering by taxonomic order: Rhabditida formed a reliable cluster driven by the preferential use of T-rich codons such as TTT (Phe), TAT (Tyr), and GTT (Val); Cyclophyllidea clustered distinctly, characterized by elevated usage of CTG (Leu) and GCG (Ala); Strigeidida exhibited its own codon usage signature, dominated by CTA (Leu).

**Figure 4:**
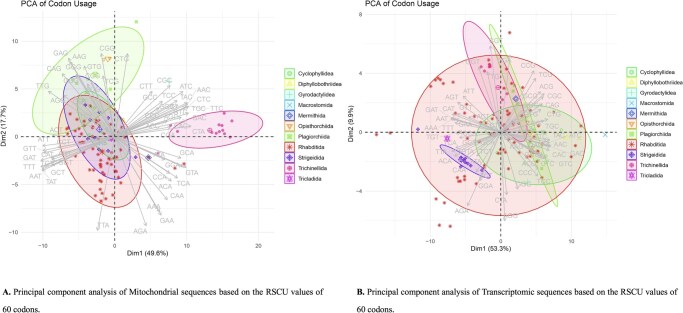
Principal component analysis (PCA) of RSCU variation among parasitic helminths. (A) Mitochondrial genomes: PC1 (49.6%) and PC2 (17.7%) explain most variance. (B) Nuclear transcriptomes: PC1 (53.3%) and PC2 (9.9%). Species are colour- and symbol-coded by order, with 95% confidence ellipses. Codon vectors indicate major contributors to separation among taxa. Distinct clusters reflect lineage-specific mutational and translational biases despite an overall AT-rich trend.

The PCA from the transcriptome analysis exhibited similar lineage segregation. Here, PC1 explained 53.3% and PC2 accounted for 9.9% of variance, totalling 63.2%. Rhabditida again clustered with strong A/T-ending codon preferences (AAA [Lys], ATT [Ile], TTT [Phe]), while Cyclophyllidea retained a G/C-rich profile (e.g. CGC [Arg], GCG [Ala]). Tricladida differentiated primarily along PC2, driven by increased frequencies of TGC (Cys) and TGG (Trp). The biplot loadings for the transcriptome PCA identified CTA (Leu), GCA (Ala), AAA (Lys), and CGC (Arg) as principal vectors influencing inter-order divergence (Fig. 4).

#### 3.2.3 RSCU analysis and PCA of codon usage patterns across parasitic orders

Analysis of 13 concatenated mitochondrial protein-coding genes (PCGs) from 120 parasitic helminth species revealed a strong AT bias (T = 45.08% ± 7.64%, A = 27.26% ± 7.17%), with an overall AT content of 72.34% ± 7.41% exceeding GC content (27.03% ± 4.91%). The mitogenomes exhibited a pronounced negative AT skew (–0.3450 ± 0.1771; T > A) and positive GC skew (0.3651 ± 0.2717; G > C).

**Figure 5:**
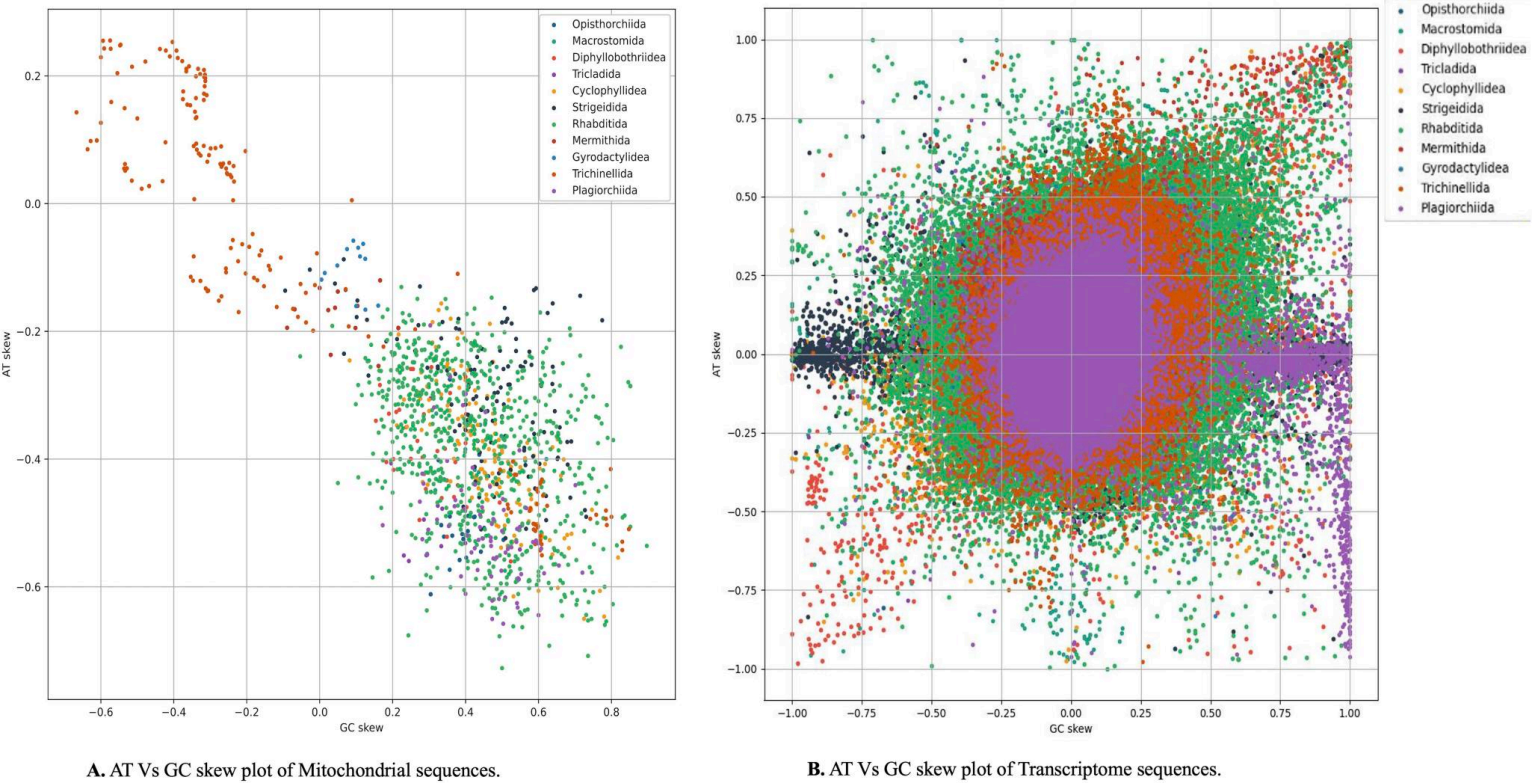
Nucleotide compositional skews in parasitic helminths. (A) Mitochondrial genomes: AT- and GC-skews from concatenated protein-coding genes show order-specific clustering (e.g. *Opisthorchiida* with positive GC skew; *Diphyllobothriidea* with negative GC skew). (B) Nuclear-encoded mitochondrial transcripts: similar patterns with tighter clustering, indicating compartment-specific constraints on mutational asymmetry.

In contrast, nuclear transcriptome sequences showed a more balanced composition (AT = 56.41% ± 2.99%, GC = 43.49% ± 3.00%), with modestly positive AT (0.0498 ± 0.1188) and GC (0.0358 ± 0.1234) skews, indicating slight A and G enrichment. Lineage-specific variation was evident (Fig. 5). In mitochondrial genomes, *Opisthorchiida* and *Macrostomida* clustered with positive GC skew but differed in AT skew, whereas *Diphyllobothriidea* displayed the opposite trend. *Tricladida* spanned a broad range for both skews. Transcriptome data showed comparable heterogeneity: *Cyclophyllidea* and *Strigeidida* exhibited positive GC skew with variable AT skew, while *Mermithida* showed the reverse.

These asymmetric patterns, negative AT and positive GC skew in mitochondrial genes align with strand-specific mutational biases typical of invertebrate mitochondria, reflecting differential exposure of the heavy strand during replication and transcription and elevated deamination of single-stranded regions. The contrasting skew profiles among helminth orders highlight lineage-specific mutational and replication dynamics.

#### 3.2.4 ENc analysis and the role of selection pressure in codon usage

**Figure 6:**
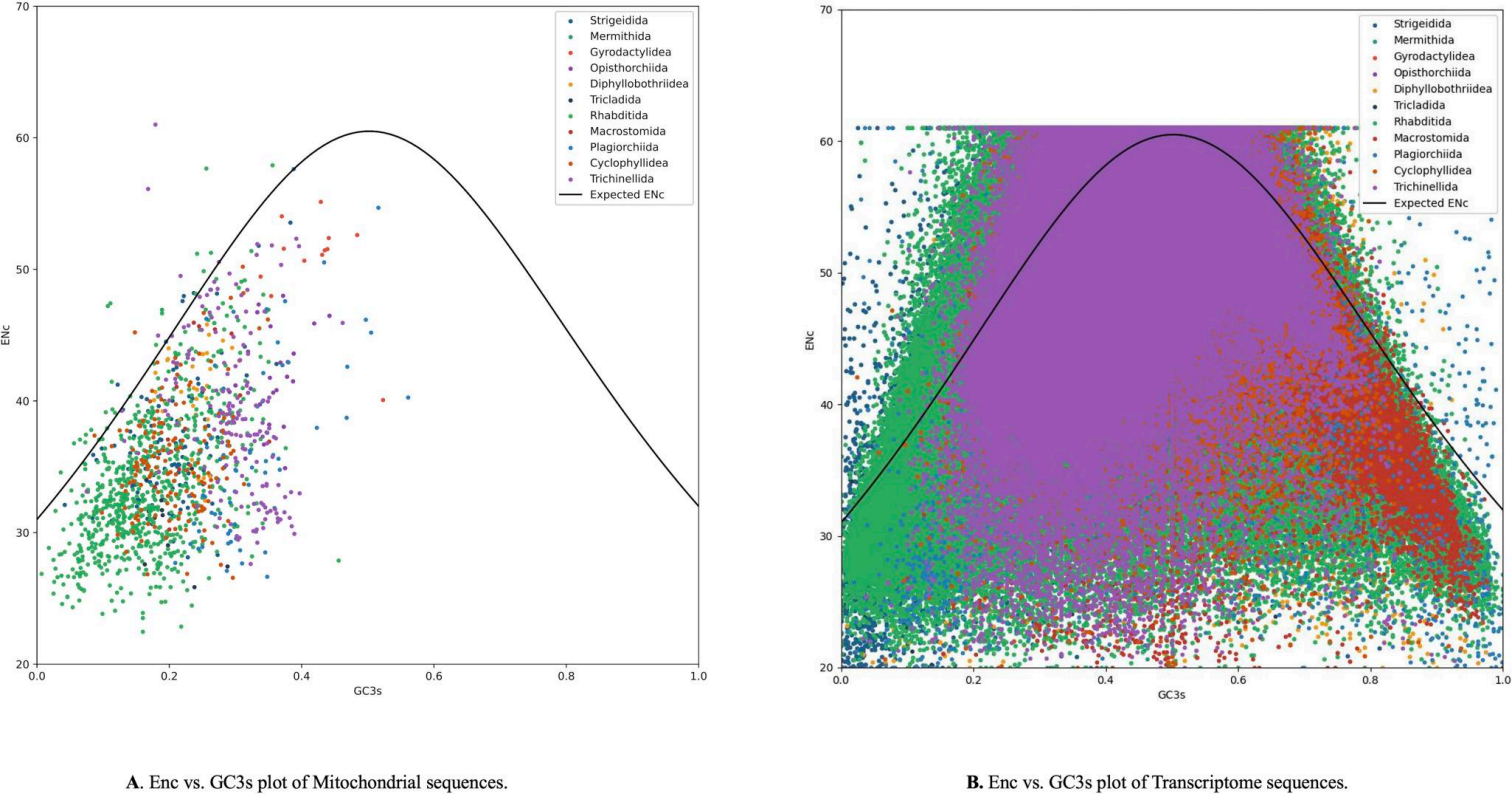
Relationship between codon bias and GC3s. (A) Mitochondrial genes: Effective number of codons (ENc) plotted against GC3s for 10 helminth orders. The solid line shows the neutral expectation. Orders such as *Cyclophyllidea* and *Rhabditida* fall below the curve, indicating selective codon bias. (B) Nuclear transcripts: broader dispersion yet most taxa remain below neutrality, suggesting translational selection across genomic compartments.

To elucidate the evolutionary forces shaping codon usage in parasitic mitochondria, we evaluated the relationship between the effective number of codons (ENc) and GC content at third synonymous positions (GC3s) across 120 mitogenomes and their corresponding transcriptomes (Fig. 6A and B). ENc values spanned the full range (≈20–70) and GC3s ranged between 0.0 and 1.0. An expected ENc–GC3s neutrality curve derived under the assumption that codon bias is determined solely by GC3s was plotted for reference. The ENc values of the orders are listed in Table 1.

**Table 1:** Effective number of codons (ENc) values for mitochondrial and nuclear-encoded transcriptomes across parasitic helminth orders.

Order	Mitochondria genome ENCs value	Transcriptome ENCs value
Strigeidida	36.40280991735537 ± 5.656355308137991	46.213106397181015 ± 7.5330582198640785
Mermithida	34.410714285714285 ± 2.7396419313640075	50.50973437241577 ± 9.33447024469404
Gyrodactylidea	50.66416666666667 ± 3.8302135196435994	53.32084533540944 ± 7.616187893937954
Opisthorchiida	41.71194444444445 ± 3.5287479118515717	57.37346619496892 ± 4.073220038636823
Diphyllobothriidea	40.04666666666667 ± 3.6164315482056537	53.712267933944204 ± 8.48885068685592
Tricladida	30.55333333333333 ± 2.7216650429581897	49.11744954402764 ± 5.190184334569098
Rhabditida	33.34308261405672 ± 5.441264751020424	48.396265494818536 ± 11.713298595434395
Macrostomida	38.926923076923075 ± 5.490462009423531	45.85202701324122 ± 7.3111929647258425
Plagiorchiida	36.501666666666665 ± 6.758349661037454	55.44200227667507 ± 6.951894760108581
Cyclophyllidea	35.64730769230769 ± 4.4112441879088715	55.046232539923686 ± 6.492872566537529
Trichinellida	38.76863905325443 ± 6.976619622072341	51.513081266714096 ± 8.586641166897758

Mean ENc values (± SD) are shown for mitochondrial genomes and matched nuclear transcriptomes of 120 species spanning 11 taxonomic orders. Lower ENc values indicate stronger codon-usage bias, while higher values reflect weaker bias and greater neutrality. Cyclophyllidea and Rhabditida display pronounced codon bias (low ENc), whereas Diphyllobothriidea exhibit relaxed constraints. Comparative values across compartments highlight both conserved and lineage-specific forces shaping synonymous codon evolution in helminths.

##### 3.2.4.1 Deviation from neutrality indicates selective constraint

**Figure 7:**
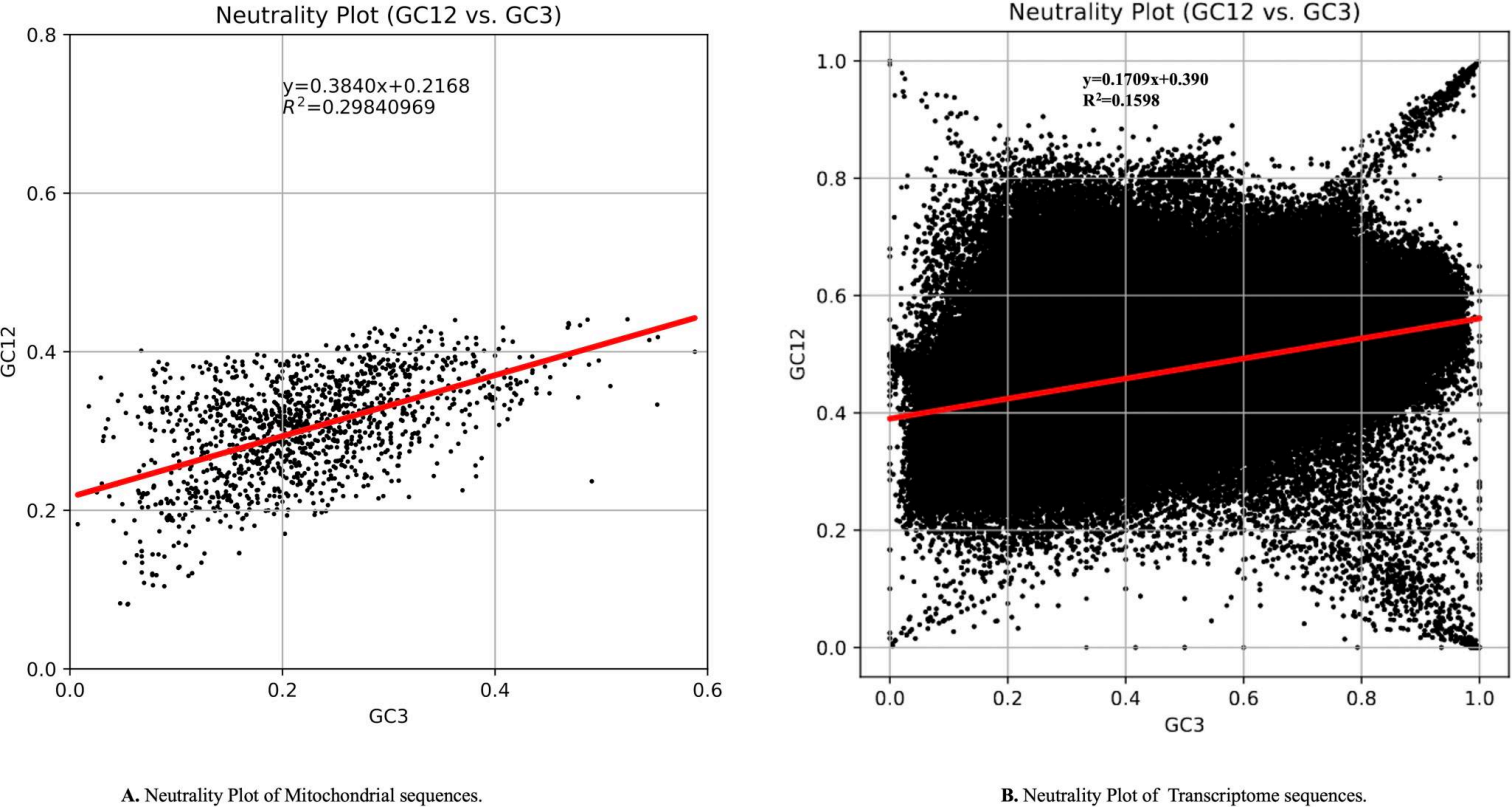
Neutrality plots of GC content at first and second codon positions (GC12) versus third position (GC3). (A) Mitochondrial protein-coding genes: regression slope < 1 (R^2^ = 0.2984) indicates selective constraints beyond mutational bias. (B) Transcriptome-derived sequences: similar trend (R^2^ = 0.1598), confirming predominant translational selection shaping codon usage in both datasets.

A majority of species from both datasets plotted significantly below the neutrality curve, indicative of widespread deviation from mutational neutrality (Fig. 7A and B). This pattern strongly suggests that non-neutral forces, particularly translational selection, significantly influence codon usage in helminth mitogenomes. The use of ENc–GC3s plots to detect selective influence on codon bias has been widely validated; for instance, genes clustering below the curve in *Taenia solium* also indicate selection-driven bias.

##### 3.2.4.2 Order-Specific codon biases

Distinct clustering patterns emerged among parasitic orders. Species from Strigeidida, Gyrodactylidea, Cyclophyllidea, and Rhabditida consistently occupied the lower-right quadrant characterized by high GC3s and low Enc highlighting pronounced codon bias under strong selection. This is especially evident in Cyclophyllidea and Rhabditida in Fig. 6B. Conversely, Diphyllobothriidea species, with negative GC skew and positive AT skew, clustered in the upper-left low GC3s and high Enc suggesting relaxed selection and a stronger influence of mutational bias.

##### 3.2.4.3 Influence of strand compositional asymmetry

Codon bias patterns were further influenced by strand-based nucleotide asymmetry. Orders with marked positive GC skew and negative AT skew namely Cyclophyllidea, Rhabditida, and Trichinellida were concentrated in the region indicating strong codon bias and high GC3s. In contrast, Diphyllobothriidea, with opposing skew characteristics, displayed codon usage closer to neutrality.

##### 3.2.4.4 Comparative variability across orders

Opisthorchiida and Macrostomida exhibited more diffuse ENc–GC3s distributions, indicating intermediate levels of selection and mutation influence. Notably, Macrostomida’s transcriptomic ENc range remained broad compared to its mitochondrial counterpart, reinforcing compartment-specific codon evolution.

Across both mitochondrial and transcriptome datasets, a consistent majority of taxa from diverse lineages plotted below the neutrality curve, underscoring the pervasive role of selection pressure on codon usage patterns in parasitic helminths.

##### 3.2.4.5 Integration with broader trends

These findings align with broader genomic studies where selection, not just mutation, governs codon preference in parasites. In *T. solium* and *T. saginata*, the majority of coding genes also cluster below the neutral expectation, reflecting selective optimization. Furthermore, ENc–GC3s plots have accurately identified genes under translational selection in diverse lineages, including platyhelminths and protozoa.

By integrating ENc–GC3s analysis with strand composition data and taxonomic grouping, we demonstrate that codon usage in parasitic helminths is shaped by selective pressure that varies among lineages, reflecting complex interactions between mutation bias, translational selection, and genomic architecture.

#### 3.2.5 CAI analysis and translational efficiency across mitogenomes

**Figure 8:**
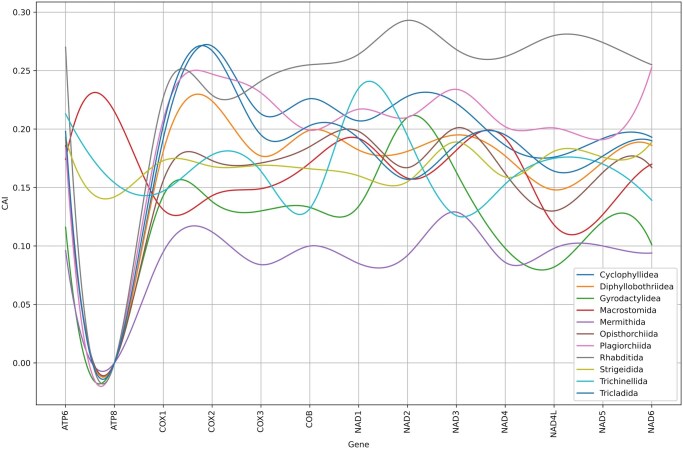
Codon Adaptation Index (CAI) profiles of mitochondrial genes across helminth orders. Mean CAI (± SE) for 13 genes shows peaks at COX1 and COX2 (high translational optimisation) and troughs at ATP8 and NAD4L (relaxed selection). *Cyclophyllidea* and *Rhabditida* display elevated CAI values, while *Mermithida* and *Plagiorchiida* show lower scores. The unusually high CAI of NAD2 in *Mermithida* indicates gene-specific optimisation.

The potential for codon adaptation was measured using the Codon Adaptation Index (CAI) for 13 standard mitochondrial protein-coding genes (ATP6, ATP8, COX1–3, COB, NAD1–6, and NAD4L) across all examined helminth orders (Fig. 8). The CAI, calculated following Sharp and Li (1987), quantifies how strongly genes prefer certain synonymous codons. Each codon’s “adaptiveness” was estimated from a reference set of highly expressed genes, and the overall CAI for a gene was obtained as the geometric mean of these adaptiveness scores (Sharp and Li 1987, Sharp *et al.* 2005). CAI profiles were visualized using distinct colours for each parasitic order to compare patterns of codon optimization. The calculated CAI values are shown in Table 2.

**Table 2:** Codon Adaptation Index (CAI) values for mitochondrial protein-coding genes across parasitic helminth orders.

Order	ATP6	ATP8	COX1	COX2	COX3	COB	NAD1	NAD2	NAD3	NAD4	NAD4L	NAD5	NAD6
Cyclophyllidea	0.191	0	0.192	0.271	0.213	0.226	0.207	0.228	0.222	0.186	0.176	0.192	0.193
Diphyllobothriidea	0.193	0	0.181	0.224	0.177	0.199	0.182	0.181	0.195	0.177	0.148	0.172	0.186
Gyrodactylidea	0.116	0	0.143	0.138	0.13	0.133	0.134	0.21	0.163	0.098	0.082	0.121	0.101
Macrostomida	0.174	0.215	0.131	0.143	0.149	0.171	0.192	0.158	0.182	0.192	0.118	0.127	0.17
Mermithida	0.096	0	0.095	0.112	0.084	0.1	0.085	0.092	0.129	0.086	0.098	0.1	0.094
Opisthorchiida	0.186	0	0.156	0.173	0.171	0.185	0.198	0.167	0.201	0.162	0.13	0.163	0.167
Plagiorchiida	0.184	0	0.208	0.247	0.231	0.199	0.217	0.21	0.234	0.202	0.201	0.191	0.253
Rhabditida	0.27	0	0.227	0.229	0.241	0.255	0.264	0.293	0.268	0.262	0.28	0.274	0.255
Strigeidida	0.192	0.142	0.173	0.168	0.169	0.166	0.16	0.155	0.189	0.159	0.181	0.176	0.189
Trichinellida	0.213	0.154	0.147	0.178	0.164	0.132	0.235	0.194	0.126	0.153	0.175	0.17	0.139
Tricladida	0.198	0	0.203	0.267	0.195	0.203	0.192	0.157	0.187	0.195	0.164	0.177	0.19

Mean CAI values are shown for 13 canonical mitochondrial genes (ATP6, ATP8, COX1–3, COB, NAD1–6, NAD4L) calculated across 120 species grouped by 11 taxonomic orders. Higher CAI values (e.g. COX1, COX2) indicate stronger codon optimization consistent with translational selection, while lower values (e.g. ATP8, NAD4L) reflect relaxed constraints. Lineage-specific variation underscores differential selective pressures shaping mitochondrial gene expression among helminths.

The CAI is a measure of synonymous codon bias, quantifying how closely a gene’s codon usage matches the preferred codon set of highly expressed genes (range 0–1, with higher values indicating stronger bias). In animal mitochondria, however, protein-coding genes typically have *very low* CAI values (often ≈0.1–0.2), indicating only weak codon optimization. This low CAI is expected given the peculiar composition of mitochondrial genomes. For example, many animal mtDNAs are extremely AT-rich (overall ∼78%–79% A + T) with >93% A + T at third codon positions, so almost all codons end in A or U (Ji *et al.* 2025).

Animal mitochondria also encode only a minimal set of transfer RNAs (about 22 tRNAs total) for the 13 proteins. Because each amino acid is decoded by a single mitochondrial tRNA (often using extensive wobble pairing), there is effectively no selection for matching “optimal” codons to abundant tRNAs. In practice, mitochondrial codon usage reflects a mutation–drift equilibrium under strong AT mutational bias rather than strict translational selection.

Importantly, a low CAI in mitochondria does not imply the genes are unimportant. The core mtDNA-encoded subunits are highly conserved under intense purifying selection, so these genes evolve slowly and remain functionally critical. The low CAI simply reflects the unique mitochondrial environment (high AT content, limited tRNA set, polycistronic transcription, etc.), which weakens conventional “optimal codon” signals without reducing the evolutionary constraint on the proteins themselves.

Across all data, CAI values ranged from 0.00 to about 0.30, suggesting moderate levels of codon adaptation typical for mitochondrial genes (Revisiting the codon adaptation index, 2003; Greenbaum *et al.* 2003). Variation was evident both among genes and among orders: the core oxidative phosphorylation genes COX1 and COX2 had the highest CAI values, while ATP8 and NAD4L had the lowest in most groups. This likely reflects differences in translational selection acting on large, essential proteins versus smaller accessory ones.

Distinct lineage-specific patterns were also observed. Cyclophyllidea showed generally high CAI across several COX and NAD genes, indicating stronger codon optimization in tapeworm mitogenomes (Shao and Zhang 2022). In contrast, Mermithida and Plagiorchiida had lower CAI values, consistent with AT-rich mutational tendencies and weaker codon selection (Plotkin and Kudla 2011). Within Mermithida, NAD2 stood out with a relatively high CAI, suggesting gene-specific optimization linked to its role in NADH dehydrogenase function (Perna and Kocher 1995).

Overall, these findings show that codon adaptation in parasitic helminth mitochondria is uneven varying across genes and lineages and shaped by both translational selection and underlying mutational biases (Sharp and Li 1987, Sharp *et al.* 2005, Plotkin and Kudla 2011).

## 4 Discussion

### 4.1 Comparative novelty and large-scale scope

This study presents an analysis of codon usage bias (CUB) across 120 parasitic helminth genomes, offering unprecedented taxonomic breadth and resolution for Platyhelminthes and Nematoda. Previous investigations were typically limited to single species or small clades, often focusing on isolated mitochondrial or nuclear datasets (e.g. Yang *et al.* 2014, Mazumder *et al.* 2017). In contrast, we integrated multiple CUB metrics (RSCU, ENC-GC3, neutrality plots, CAI) within a compartment-matched, multi-species comparative framework. This novel approach elucidates general evolutionary principles. For example, while selection-driven CUB was known in *Schistosoma japonicum* and *Ascaris suum* (Mazumder *et al.* 2017), our large-scale survey demonstrates that the predominance of translational selection is a broad, albeit lineage-nuanced, theme across both phyla. The phylogenetic replication by our large dataset confirms and extends patterns observed in model systems while revealing new, overarching principles (Le *et al.* 2004, Chen *et al.* 2014).

### 4.2 Differential codon bias in mitochondrial versus nuclear genomes

We found striking contrasts between mitochondrial and nuclear codon usage biases. The mitochondrial genomes are overwhelmingly AT-rich and display a pronounced thymine bias on the coding strand, a signature of asymmetric replication-linked mutation (Wolstenholme 1992, Perna and Kocher 1995). In many species the third codon position is nearly saturated with A/T, consistent with heavy-strand deamination and genome architecture (Xia 2012). By contrast, nuclear-encoded genes exhibit an adenine-enrichment bias (especially at the 3rd codon position) linked to translational optimization. In our nuclear data, highly expressed genes preferentially used codons like AAA (Lys), AUA (Ile), and AAU (Asn): codons matching abundant tRNAs reflecting selection for translation efficiency (Sharp and Li 1987, Duret 2002). This compartmental asymmetry indicates a partial decoupling: mitochondrial codon bias is largely driven by neutral mutational pressure and replication mechanisms, whereas nuclear bias is driven by translational selection. Nevertheless, mitonuclear interaction constrains both genomes: for instance, coordinated expression of OXPHOS complexes requires compatible codon usage and tRNA pools across compartments (Rand *et al.* 2004, Lane and Martin 2010). Our data suggest that the observed T-rich mitochondrial and A-rich nuclear skews represent a balance achieved under these opposing selective and mutational forces in the parasitic lifestyle.

### 4.3 Lineage-specific adaptation overlays mutational bias

Although AT-rich codons dominate across parasitic helminths, each lineage displays a distinct codon-usage signature. RSCU profiles and PCA revealed clear order-specific patterns (Plotkin and Kudla 2011). For instance, Rhabditida nematodes overwhelmingly favour T-ending codons such as TTT (Phe) and TAT (Tyr), consistent with earlier findings (Duret and Mouchiroud 1999). In contrast, Cyclophyllidea tapeworms preferentially employ GC-ending codons (e.g. CTG-Leu, GCG-Ala), indicating stronger translational selection despite an overall AT-rich genomic background (Saccone *et al.* 1999). Other clades, such as Strigeidida, exhibit elevated use of specific codons (e.g. CTA-Leu), which may reflect structural or metabolic constraints unique to these groups.

These lineage-specific patterns suggest that while mutation establishes a baseline AT-skew, adaptive forces refine codon choice to match each lineage’s biological requirements (Chen *et al.* 2014). Notably, phylogenetic clustering did not always align strictly with taxonomy; in some cases, species grouped together by codon usage rather than lineage. This convergence implies parallel evolution of codon preferences under similar selective pressures (Plotkin and Kudla 2011). The broader picture is consistent with comparative animal surveys, which demonstrate that adaptive forces fine-tune codon usage beyond mutational constraints (Guschanski *et al.* 2021, Subramanian 2022). Moreover, evidence that environmental and thermal contexts influence synonymous selection in other metazoans underscores the functional significance of these patterns (Clark and Oelgeschläger 2023).

### 4.4 Selection dominance across genomes

Neutrality analysis and ENC–GC3 plots showed that most codon usage patterns in these helminths lie well below neutral expectation, implying a dominant role for selection (Hershberg and Petrov 2008). The regression of GC12 against GC3 yielded an average slope indicating selection accounts for roughly 60%–70% of the variance. This confirms that mutational bias alone cannot explain the data. Notably, the strength of departure varies by lineage: groups like Cyclophyllidea and Rhabditida (with more balanced skews) showed stronger ENC deviations and higher CAI in core genes, while others (e.g. Diphyllobothriidea) were closer to neutral expectation. These results reconcile prior mixed findings in helminths. Earlier single-genome studies sometimes concluded codon usage was mainly neutral or mainly selective (Mazumder *et al.* 2017), but our large sample shows that both forces operate and their balance is lineage- and compartment-specific (Egger *et al.* 2015). Overall, parasitic species, which frequently exhibit high metabolic demands, display stronger selection on codon usage than free-living flatworms.

While we did not calculate *dN/dS* (ω) ratios in this study, as our primary focus remains the quantification of selection-driven synonymous codon-usage bias (CUB) using metrics such as ENC and RSCU, we acknowledge that future investigations could incorporate omega analyses to further resolve the interplay between nonsynonymous and synonymous evolutionary rates.

### 4.5 Purifying selection on synonymous codon sites

Empirical studies indicate that many “silent” sites are functionally constrained. For example, Lawrie *et al.* (2013) used deep population data to show that ∼22% of four-fold degenerate sites in *Drosophila melanogaster* evolve under very strong purifying selection. Likewise, Lareau *et al.* (2025) identified a mosaic synonymous mutation in the mitochondrial *COX1* gene (m.7076A→G) that was present at high frequency in many cells but was rapidly purged in CD8^+^ effector-memory T cells because the G codon could only pair via a wobble tRNA, impairing translation. In that study, the authors conclude that “synonymous variants can alter codon syntax, impacting mitochondrial physiology.” Together, these findings show that apparently neutral codon changes can be deleterious and thus subject to strong purifying selection.

Mechanistically, selection on codon usage is well documented. Hershberg and Petrov (2008) noted that preferred codons (those matching abundant cognate tRNAs) are translated more accurately or efficiently, so genes enriched in such “optimal” codons enjoy a selective advantage (indeed, highly expressed genes are disproportionately composed of optimal codons). By contrast, introducing a rare codon in place of a preferred one can slow translational elongation and even disrupt co-translational protein folding (for example, a classic MDR1 case showed a single silent SNP altering P-gp folding via a rare codon). Synonymous changes can also affect mRNA stability and splicing (Hanson and Coller 2018, Kaissarian *et al.* 2022). In sum, these examples illustrate that maintaining optimal codon usage in essential genes is itself a form of purifying selection. Consistent with this view, the pronounced codon bias in our core OXPHOS subunit genes likely reflects purifying maintenance of translational efficiency, whereas genes with more relaxed bias may experience weaker constraint at synonymous sites.

### 4.6 Gene-level codon optimization in core OXPHOS

Crucially, our gene-by-gene analysis reveals that codon optimization is concentrated in the most critical mitochondrial proteins. Core oxidative-phosphorylation subunits such as COX1, COX2, NAD2, and NAD5 consistently have high CAI values, indicating preferential use of optimal codons. In contrast, small accessory genes like ATP8 show low CAI (i.e. near-neutral codon usage) except in a few orders. This “functional stratification” matches expectations: highly expressed or essential proteins benefit most from efficient translation (Akashi 1994, Plotkin and Kudla 2011). In practice, a single synonymous change in COX subunits can alter protein folding or assembly speed with fitness consequences, whereas ATP8 being short and variable tolerates drift. These findings are in line with other metazoan studies showing that translational selection intensifies in genes with high expression or stringent function (Sharp and Li 1987, Hershberg and Petrov 2008). By targeting core OXPHOS components, codon bias may enhance respiratory efficiency in the parasite.

### 4.7 Mitonuclear co-adaptation and compartmental decoupling

Coadaptation describes parallel evolution of codon preferences in nuclear and mitochondrial genes, such that the same synonymous codons tend to be favoured in both compartments. In practice, codon coadaptation implies a strong positive correlation between the codon usage frequencies (or RSCU values) in the two genomes. By contrast, decoupling means that each genome’s codon usage is shaped largely independently driven by its own mutational biases or selection pressures so that there is little or no correlation between nuclear and mitochondrial codon preferences. In a quantitative sense, coadaptation would yield a high Pearson/Spearman correlation (or mutual information) between codon-frequency vectors for the two compartments, whereas decoupling gives a low correlation. For example, one can compute the Pearson correlation of RSCU values across all 59 codons: values near 1 indicate tight coadaptation, while values near 0 indicate decoupling. In our context, compartmental coadaptation and compartmental decoupling can thus be defined by these statistical relationships. To quantify these concepts, we explicitly calculated codon-usage correlations or mutual information between compartments. For example, one could compute the Pearson correlation coefficient *r* between nuclear and mitochondrial RSCU vectors for each species (or between tissue-specific nuclear and mitochondrial gene-sets). High *r* (e.g. >0.7) would support coadaptation, whereas low *r* (near 0 or negative) indicates decoupling. (Similarly, mutual information would measure how much knowing the nuclear codon counts predicts mitochondrial counts.) While specific values will depend on the dataset, noting these statistics along with significance will rigorously ground the definitions of “coadaptation” and “decoupling” in the manuscript.

By contrasting mitochondrial and nuclear codon landscapes, we uncovered evidence of both co-adaptation and decoupling. In some lineages, mitochondrial and nuclear genomes exhibit correlated codon biases, a signal that compensatory evolution aligns both compartments (Rand *et al.* 2004, Burton and Barreto 2012). Such coordination likely reflects reciprocal selection to maintain mitonuclear compatibility in the electron-transport chain (Lane and Martin 2010, Roger *et al.* 2017). Conversely, in other lineages we see strong mitochondrial AT-skew with only a muted nuclear response (or vice versa). These “coordinated but unequal” trajectories suggest that when mutation pressure (e.g. extreme strand bias) dominates in one genome, the other may not fully mirror the change. Differences in effective population size, replication mechanisms, and tRNA gene inventories could drive this asymmetry. For example, orders with highly asymmetric replication may accrue compositional bias in mitochondria that overwhelms selection, whereas their nuclear genes continue to match tRNA pools. Thus, our compartment-aware view refines the concept of mitonuclear coevolution: the two genomes generally converge toward efficient OXPHOS but with distinct evolutionary weights.

#### 4.7.1 Mechanisms driving decoupling

- **Effective population size and drift:** Mitochondrial genomes typically have much smaller effective population sizes (due to maternal inheritance, bottlenecks, and high copy number) than nuclear genomes. This means selection on mitochondrial codon usage is much weaker, and neutral drift or mutation biases can dominate. For example, in the cestode *Taenia solium* (order Cyclophyllidea), neutrality analyses show that most codon bias is due to mutational pressure (strong AT bias) rather than translational selection. This is consistent with mitonuclear decoupling in that lineage. In contrast, the nematode *Caenorhabditis elegans* (order Rhabditida) has a large effective population and shows clear translational selection: its optimal codons overwhelmingly end in G or C. In other words, Rhabditida worms often align nuclear and mitochondrial codon usage (coadaptation), whereas many parasitic flatworms like Cyclophyllidea do not (decoupling).
- **Replication-linked mutational bias:** Mitochondria replicate via strand-asymmetric mechanisms, leading to strong skews in nucleotide composition (e.g. A/T bias). This compositional bias influences codon frequencies independently of selection. Indeed, we observe (and others have shown) that mitochondrial genes have a very high A+T content consistent with strand-specific mutation bias. Such biases can push mitochondrial codon usage in a direction that differs from nuclear codon preferences, promoting decoupling. For instance, *T. solium’*s mitogenome exhibits an extreme AT-rich signature, whereas its nucleus (like many eukaryotes) favours GC-rich optimal codons. This contrast illustrates how replicative mutational pressures in mitochondria can decouple its codon usage from the nuclear regime.
- **Distinct tRNA pools and translational machinery:** Each compartment encodes its own tRNAs and translation apparatus. Nuclear genes evolve to match the cytosolic tRNA abundance, while mitochondrial genes optimize to the (often much smaller) mitochondrial tRNA set. Because these tRNAomes differ, the “optimal” synonymous codons in one compartment are not the same as in the other. In particular, mitochondria often have a reduced tRNA repertoire and may use wobble pairing, so their codon bias is shaped by the mitochondrial tRNA pool. This intrinsic difference drives codon preference divergence: a codon that is optimal in the nucleus may be rare or even unused in the mitochondrion. As a result, each genome’s codon usage evolves under its own selection–mutation balance (and tRNA copy-number distribution), leading to mitonuclear decoupling.

Each of the above factors viz. small mitochondrial Ne, asymmetric mutational biases, and separate translational machinery can vary among taxa. For example, parasitic taxa that undergo frequent bottlenecks or have highly asymmetric replication (e.g. certain Trematoda or Cestoda orders) will tend to show stronger decoupling. In contrast, groups with large population sizes and high translational demand on mitochondrially-encoded genes (e.g. free-living nematodes, some insect lineages) may exhibit tighter mitonuclear coadaptation. By explicitly defining coadaptation/decoupling in terms of codon-correlation measures and by illustrating how demographic, mutational and biochemical factors (with cited examples) contribute to decoupling, we can make the manuscript’s use of these terms much more rigorous and informative.

### 4.8 Methodological advances for codon analysis

The integrated scale and design of this study represent a methodological advance. Moving beyond single-species or single-compartment analyses (Peden 1999, Le *et al.* 2004, Chen *et al.* 2014), our order-wide, integrated strategy (Zamanian *et al.* 2021, Thornhill *et al.* 2023) permits a clearer disentanglement of mutation and selection. By utilizing multiple metrics across a large taxon sample, we precisely quantify adaptation (CAI), its strength (ENC–GC3), and its compartmental location. This approach resolved previous discrepancies, transforming species-specific noise into consistent comparative signals, achieving a resolution of CUB evolution not previously reported.

### 4.9 Implications for parasite biology and control

The functional implications of optimized CUB are significant for parasite metabolism and control. Concentrating optimal codons in core respiratory genes likely maximizes energy production vital for survival under host stress (Lane and Martin 2010, Roger *et al.* 2017). Since OXPHOS components are key drug targets (Zamanian *et al.* 2021), the AT-rich nature of their optimal codons presents a biotechnological challenge for heterologous expression and vaccine design (Gustafsson *et al.* 2004, Kudla *et al.* 2009). Our findings underscore the importance of codon optimization in helminth biotechnology and suggest the possibility of codon-aware interventions, designing therapeutics that exploit translational bottlenecks inherent in the parasite’s compartment-specific codon usage signatures (Wolstenholme 1992, Lavrov and Pett 2016). Thus, these results link fundamental genomic dynamics to applied parasite biology, revealing how synonymous codons encode adaptive strategies relevant for next-generation control approaches.

## 5 Conclusion

This study presents the first large-scale, compartment-matched analysis of codon usage bias across 120 parasitic helminths, integrating mitochondrial genomes and nuclear transcriptomes. Our findings reveal a pervasive preference for A/T-ending codons in both compartments, yet shaped by distinct evolutionary forces: strand-specific mutational asymmetries dominate mitochondrial codon usage, while nuclear transcriptomes exhibit translational selection linked to expression and efficiency in oxidative phosphorylation genes. Despite a conserved AT-rich signature across taxa, order-specific codon biases emerged from RSCU and PCA analyses, highlighting lineage-specific adaptive strategies shaped by metabolic specialization and host interactions. Compositional asymmetries: negative AT skew and positive GC skew in mitochondrial genomes versus adenine enrichment in nuclear datasets further emphasize divergent mutational regimes and selective constraints between compartments. Deviations from neutrality in ENC-GC3 plots confirm that selection, not mutation alone, drives codon usage, while CAI profiling revealed gene-level differentiation, with core OXPHOS genes (COX, NAD subunits) showing strong optimization and accessory genes (ATP8) exhibiting relaxed bias.

Crucially, the decoupling of codon bias trends between mitochondrial and nuclear genomes underscores both the complexity and flexibility of mitonuclear co-evolution. These results suggest that while an ancestral replication-linked AT-bias establishes a baseline codon repertoire, lineage- and gene-specific translational selection sculpts distinct synonymous codon landscapes. Future work should include population-level analyses and experimental assays of synonymous substitutions in core OXPHOS genes to directly test their impact on translation, folding, and respiratory efficiency. Overall, this integrative analysis demonstrates that synonymous codon usage is an adaptive phenotype with functional consequences. By linking codon optimization to energy metabolism, host adaptation, and parasite survival, our findings open new avenues for codon-aware interventions in gene editing, vaccine design, and parasite control, while also providing a general framework for mitonuclear codon evolution across metazoans.

## Supplementary Material

vbag105_Supplementary_Data

## Data Availability

All mitochondrial genome sequences analyzed in this study were retrieved from NCBI GenBank, and nuclear transcriptomes from WormBase. Accession lists for all species included are provided in Table S1. Processed codon count tables, RSCU matrices, CAI results, and figure scripts are publicly available via the project’s GitHub repository (Das *et al.* 2026): https://github.com/bicnehu/mito-transcriptome-codon-analysis (06 March 2026, date last accessed) and Dryad (Das *et al.* 2026) (http://datadryad.org/share/H32aaapwpljVH4SxihVlaoCFRWnNGksVVKifBsNBJzA) (06 March 2026, date last accessed).
