# Supplementary material for: Synonymous but not silent: functional codon bias reveals decoupled mitonuclear evolution in parasitic worms
Source: Bioinform Adv. 2026 May 4;6(1):vbag105. doi: 10.1093/bioadv/vbag105 (PMC13187747; doi:10.1093/bioadv/vbag105)
Supplement: vbag105_Supplementary_Data [file vbag105_supplementary_data.docx]

**Table S1.** Parasitic helminth species included in this study with associated project identifiers and GenBank accession numbers for complete mitochondrial genomes. These accessions formed the basis for codon usage, compositional bias, and mitonuclear comparative analyses.

| **Organism** | **Project_id** | **Mitochondria Accession No.** |
| --- | --- | --- |
| **Dracunculus_medinensis** | PRJEB500 | NC_016019.1 |
| **Onchocerca_flexuosa** | PRJEB512 | NC_016172.1 |
| **Meloidogyne_enterolobii** | PRJNA340324 | NC_026555.1 |
| **Ascaris_lumbricoides** | PRJEB4950 | NC_016198.1 |
| **Schistosoma_spindale** | PRJEB44434 | NC_047240.1 |
| **Clonorchis_sinensis** | PRJDA72781 | NC_012147 |
| **Trichobilharzia_szidati** | PRJEB44434 | NC_036411.1 |
| **Echinococcus_granulosus** | PRJNA182977 | OR730806.1 |
| **Schistocephalus_solidus** | PRJEB527 | MW602521.1 |
| **parascaris_univalens** | PRJNA386823 | NC_024884.1 |
| **trichinella_nelsoni** | PRJNA257433 | NC_025753.1 |
| **caenorhabditis_remanei** | PRJNA248911 | NC_035243.1 |
| **angiostrongylus_costaricensis** | PRJEB494 | NC_013067.1 |
| **mesocestoides_corti** | PRJEB510 | AP017667.1 |
| **anisakis_simplex** | PRJEB496 | NC_007934.1 |
| **wuchereria_bancrofti** | PRJEB536 | NC_016186.1 |
| **angiostrongylus_cantonensis** | PRJNA350391 | OR177661.1 |
| **meloidogyne_javanica** | PRJNA340324 | OR038715.1 |
| **spirometra_erinaceieuropaei** | PRJEB1202 | OM935781.1 |
| **macrostomum_lignano** | PRJNA371498 | NC_035255.1 |
| **schistosoma_mansoni** | PRJEA36577 | NC_002545.1 |
| **dictyocaulus_viviparus** | PRJNA72587 | NC_019810.1 |
| **setaria_digitata** | PRJNA479729 | NC_014282.1 |
| **caenorhabditis_tropicalis** | PRJNA53597 | NC_025756.1 |
| **strongyloides_stercoralis** | PRJEB528 | NC_028624.1 |
| **meloidogyne_graminicola** | PRJNA411966 | NC_056772.1 |
| **dicrocoelium_dendriticum** | PRJEB44434 | NC_025280.1 |
| **syphacia_muris** | PRJEB524 | AP017697.1 |
| **meloidogyne_incognita** | PRJEB8714 | NC_024097.1 |
| **haemonchus_placei** | PRJEB509 | NC_029736.1 |
| **schistosoma_guineensis** | PRJEB44434 | MK341584.1 |
| **schistosoma_curassoni** | PRJEB519 | AP017708.1 |
| **taenia_saginata** | PRJNA71493 | PP391461.1 |
| **trichuris_muris** | PRJEB126 | NC_028621.1 |
| **nippostrongylus_brasiliensis** | PRJEB511 | NC_033886.1 |
| **parascaris_equorum** | PRJEB514 | NC_036427.1 |
| **trichuris_trichiura** | PRJEB535 | NC_017750.1 |
| **trichinella_spiralis** | PRJNA12603 | NC_002681.1 |
| **romanomermis_culicivorax** | PRJEB1358 | NC_008640.1 |
| **caenorhabditis_elegans** | PRJNA13758 | NC_001328.1 |
| **steinernema_glaseri** | PRJNA204943 | AP017466.1 |
| **teladorsagia_circumcincta** | PRJNA72569 | NC_013827.1 |
| **caenorhabditis_briggsae** | PRJNA784955 | NC_009885.1 |
| **oscheius_tipulae** | PRJNA644888 | CP059034.1 |
| **acanthocheilonema_viteae** | PRJEB1697 | NC_016197.1 |
| **meloidogyne_chitwoodi** | PRJNA666745 | NC_024097.1 |
| **hymenolepis_diminuta** | PRJEB507 | NC_002767.1 |
| **trichinella_pseudospiralis** | PRJNA257433 | NC_025749.1 |
| **dibothriocephalus_latus** | PRJEB1206 | NC_008945.1 |
| **fasciola_gigantica** | PRJNA230515 | OR123716.1 |
| **hymenolepis_microstoma** | PRJEB124 | LC102493.1 |
| **oesophagostomum_dentatum** | PRJNA72579 | NC_013817.1 |
| **aphelenchoides_besseyi** | PRJNA834627 | NC_025291.1 |
| **brugia_timori** | PRJEB4663 | AP017686.1 |
| **taenia_solium** | PRJNA170813 | BK061219.1 |
| **strongyloides_papillosus** | PRJEB525 | NC_028622.1 |
| **ancylostoma_caninum** | PRJNA72585 | NC_012309.1 |
| **rhabditophanes_kr3021** | PRJEB1297 | LC050215.1 |
| **steinernema_carpocapsae** | PRJNA202318 | NC_005941.1 |
| **paragonimus_westermani** | PRJNA454344 | NC_027673.1 |
| **meloidogyne_arenaria** | PRJNA438575 | NC_026554.1 |
| **diploscapter_coronatus** | PRJDB3143 | NC_035106.1 |
| **trichinella_t9** | PRJNA257433 | KM357420.1 |
| **gyrodactylus_salaris** | PRJNA244375 | NC_008815.1 |
| **heligmosomoides_polygyrus** | PRJEB1203 | AP017688.1 |
| **opisthorchis_viverrini** | PRJNA222628 | OZ171481.1 |
| **schistosoma_mattheei** | PRJEB44434 | AP017710.1 |
| **hydatigera_taeniaeformis** | PRJEB534 | NC_056571.1 |
| **thelazia_callipaeda** | PRJEB1205 | NC_018363.1 |
| **schmidtea_mediterranea** | PRJNA885486 | NC_022448.1 |
| **caenorhabditis_angaria** | PRJNA51225 | NC_035246.1 |
| **toxocara_canis** | PRJNA248777 | NC_010690.1 |
| **trichinella_zimbabwensis** | PRJNA257433 | NC_025755.1 |
| **heterorhabditis_bacteriophora** | PRJNA13977 | NC_008534.1 |
| **parastrongyloides_trichosuri** | PRJEB515 | NC_028620.1 |
| **litomosoides_sigmodontis** | PRJEB3075 | AP017689.1 |
| **trichinella_britovi** | PRJNA257433 | NC_025750.1 |
| **caenorhabditis_nigoni** | PRJNA384657 | KP259621.2 |
| **trichuris_suis** | PRJNA179528 | NC_017747.1 |
| **opisthorchis_felineus** | PRJNA413383 | NC_011127.2 |
| **echinococcus_oligarthrus** | PRJEB31222 | NC_009461.1 |
| **cylicostephanus_goldi** | PRJEB498 | AP017681.1 |
| **dirofilaria_immitis** | PRJEB1797 | AP017681.1 |
| **trichinella_murrelli** | PRJNA257433 | NC_005305.1 |
| **caenorhabditis_brenneri** | PRJNA20035 | NC_035244.1 |
| **ancylostoma_duodenale** | PRJNA72581 | NC_003415.1 |
| **angiostrongylus_vasorum** | PRJNA663250 | NC_018602.1 |
| **pristionchus_pacificus** | PRJNA12644 | NC_015245.1 |
| **trichinella_t6** | PRJNA257433 | KM357418.1 |
| **hymenolepis_nana** | PRJEB508 | NC_029245.1 |
| **strongylus_vulgaris** | PRJEB531 | NC_013818.2 |
| **brugia_pahangi** | PRJEB497 | AP017680.1 |
| **schistosoma_japonicum** | PRJNA520774 | ON637114.1 |
| **haemonchus_contortus** | PRJNA205202 | NC_010383.2 |
| **brugia_malayi** | PRJNA10729 | NC_004298.1 |
| **enterobius_vermicularis** | PRJEB503 | NC_056632.1 |
| **ascaris_suum** | PRJNA80881 | NC_001327.1 |
| **trichobilharzia_regenti** | PRJEB44434 | NC_009680.1 |
| **bursaphelenchus_xylophilus** | PRJEB40022 | NC_023208.1 |
| **caenorhabditis_tribulationis** | PRJEB12608 | OL362111.1 |
| **echinococcus_canadensis** | PRJEB8992 | OQ161122.1 |
| **echinostoma_caproni** | PRJEB1207 | AP017706.1 |
| **schistosoma_margrebowiei** | PRJEB44434 | AP017709.1 |
| **trichinella_nativa** | PRJNA257433 | NC_025752.1 |
| **schistosoma_haematobium** | PRJEB44434 | NC_008074.1 |
| **strongyloides_ratti** | PRJEB125 | NC_028623.1 |
| **panagrellus_redivivus** | PRJNA186477 | AP017464.1 |
| **necator_americanus** | PRJNA72135 | NC_003416.2 |
| **trichinella_papuae** | PRJNA257433 | NC_025754.1 |
| **taenia_multiceps** | PRJNA307624 | NC_013844.1 |
| **strongyloides_venezuelensis** | PRJEB530 | NC_028229.1 |
| **gongylonema_pulchrum** | PRJEB505 | NC_026687.1 |
| **taenia_asiatica** | PRJNA299871 | PP391462.1 |
| **fasciolopsis_buski** | PRJNA284521 | NC_030528.1 |
| **halicephalobus_mephisto** | PRJNA528747 | NC_085811.1 |
| **onchocerca_ochengi** | PRJEB1204 | NC_031891.2 |
| **echinococcus_multilocularis** | PRJEB122 | LC744000.1 |
| **onchocerca_volvulus** | PRJEB513 | NC_001861.1 |
| **fasciola_hepatica** | PRJEB25283 | NC_002546.1 |
| **ancylostoma_ceylanicum** | PRJNA231479 | NC_035142.1 |
